# Biomarker-Based Precision Prediction of Immunotherapy Response in Hepatocellular Carcinoma

**DOI:** 10.3390/diagnostics16010085

**Published:** 2025-12-26

**Authors:** Hsu-Wen Chao, Yi-Mei Joy Lin, Chen-Shiou Wu

**Affiliations:** 1 Department of Physiology, School of Medicine, College of Medicine, Taipei Medical University, Taipei 11031, Taiwan; chaohw3619@tmu.edu.tw; 2 Graduate Institute of Medical Sciences, College of Medicine, Taipei Medical University, Taipei 11031, Taiwan; 3 Department of Biomedical Science and Environmental Biology, Kaohsiung Medical University, Kaohsiung 80708, Taiwan; 4 Institute of Biomedical Sciences, National Chung Hsing University, Taichung 40227, Taiwan; ymjlin@nchu.edu.tw; 5 Department of Medical Research, Taichung Veterans General Hospital, Taichung 40705, Taiwan

**Keywords:** hepatocellular carcinoma, immunotherapy response prediction, metabolic gene signature, biomarker discovery, machine learning

## Abstract

**Background**: Hepatocellular carcinoma (HCC) remains a major global health challenge with limited treatment options for advanced disease. Although immune checkpoint inhibitors (ICIs) have shown clinical benefits, response rates remain low, emphasizing the need for reliable biomarkers to guide patient selection. Given the critical role of metabolic reprogramming in immune modulation, this study aimed to identify a metabolic gene signature predictive of immunotherapy response in HCC. **Methods**: Three independent transcriptomic datasets (GSE279750, GSE215011, and GSE235863) comprising 35 ICI-treated HCC samples were integrated after quality control and ComBat batch correction. Differentially expressed genes were identified using DESeq2 and limma, followed by integration of the meta-analysis results. Machine learning models, including LASSO regression and random forest algorithms, were applied for feature selection, and a logistic regression model was developed for predictive scoring. **Results**: A five-gene metabolic signature (PLPPR1, CNTN3, HOXA10, HAGLR, and ENPP3) demonstrated good discriminative ability between responders and non-responders, with consistent performance observed across internal validation analyses. Functional enrichment analysis revealed significant involvement of metabolic pathways, with HOXA10 linked to immune evasion and CNTN3 associated with immune activation. **Conclusions**: This five-gene signature represents a biologically interpretable biomarker panel with potential utility for immunotherapy response stratification in HCC. The integrative analytical framework provides preliminary evidence supporting its value, warranting further validation in larger, independent clinical cohorts before clinical translation.

## 1. Introduction

Hepatocellular carcinoma (HCC) represents a major global health challenge with limited therapeutic options for advanced disease [1,2]. While immune checkpoint inhibitors have emerged as promising treatments, clinical outcomes remain highly variable, with response rates of only 15–30% in HCC patients [3,4]. This therapeutic heterogeneity underscores the critical need for robust predictive biomarkers to guide treatment selection. Current biomarker approaches, including PD-L1 expression and tumor mutational burden, demonstrate insufficient predictive accuracy in HCC due to its unique molecular complexity and diverse etiology [5,6,7]. The intricate tumor microenvironment, characterized by chronic inflammation and metabolic dysfunction, creates distinct challenges for immunotherapy efficacy prediction [2,8].

Emerging evidence highlights the pivotal role of metabolic reprogramming in determining immunotherapy response [9,10]. Specifically, dysregulated pathways such as glycolysis, fatty acid oxidation, and amino acid metabolism have been implicated in modulating T-cell exhaustion and immune evasion in HCC [11,12]. These metabolic dependencies represent an underexplored dimension for biomarker development. Advanced computational approaches, particularly machine learning techniques combining Least Absolute Shrinkage and Selection Operator (LASSO) regression and random forest algorithms, offer powerful methodologies for identifying clinically relevant gene signatures from high-dimensional transcriptomic data [13,14]. Such approaches can effectively handle complex genomic datasets while minimizing overfitting risks. This study integrates transcriptomic profiles from three independent HCC cohorts receiving immune checkpoint inhibitor therapy. To address this unmet need, we aimed to identify robust metabolic gene signatures predictive of immunotherapy response in HCC by integrating multi-cohort transcriptomic data and applying advanced machine learning models. Through systematic application of meta-analysis, LASSO regularization, and random forest feature selection, we identified a five-gene metabolic signature (PLPPR1, CNTN3, HOXA10, HAGLR, ENPP3) that accurately distinguishes treatment responders from non-responders. This integrative approach may facilitate patient stratification and guide precision immunotherapy in clinical practice.

## 2. Materials and Methods

### 2.1. Data Acquisition and Preprocessing

The three Gene Expression Omnibus (GEO) datasets [15] were selected to represent independent and clinically relevant cohorts of HCC patients treated with immune checkpoint inhibitors. GSE279750 (*n* = 10) includes patients receiving first-line anti–PD-L1–based combination immunotherapy, with surgical tumor specimens collected after more than three months of treatment and classified as responders or non-responders according to modified RECIST criteria [5]. GSE215011 (*n* = 10) comprises tumor RNA-seq data from patients treated with nivolumab monotherapy (anti–PD-1), enabling comparison of transcriptional profiles between responders and non-responders [16]. GSE235863 (*n* = 15) represents HBV-positive HCC patients receiving anti–PD-1 plus lenvatinib combination therapy (pembrolizumab or sintilimab) and includes paired samples collected before or after treatment initiation; responders were defined as patients achieving complete or partial response (CR/PR), whereas non-responders were defined as those with stable or progressive disease (SD/PD) [17]. All datasets were generated using next-generation sequencing–based transcriptomic profiling and provided clinical response annotations. Across the three datasets, a total of 35 patients were included, comprising 22 responders and 13 non-responders based on the original clinical annotations. For consistency, only tumor-derived gene expression data with available response information were included in the integrative analysis. Raw count data underwent quality control, excluding samples with low sequencing depth (<1 million reads) or excessive missing values (>20%). Gene expression values were log2-transformed after adding a pseudocount of 1. Principal component analysis (PCA) was performed using the prcomp() function in R (https://www.R-project.org/) with center = TRUE and scale = TRUE parameters to visualize sample distribution and assess separation between response groups.

### 2.2. Batch Effect Correction and Data Integration

The ComBat algorithm from the sva package (version 3.50.0) was applied for batch effect correction. The ComBat() function implemented parametric empirical Bayes adjustments, treating dataset origin as the batch variable and treatment response as the protected biological variable. This approach removed technical variation while maintaining biological differences between groups. Post-correction quality was confirmed through integrated principal component analysis. The ComBat-corrected expression matrix served as the foundation for subsequent analyses.

### 2.3. Differential Expression Analysis

Individual dataset analyses were conducted using DESeq2 (version 1.42.0) with default parameters. Genes with absolute log_2_ fold-change greater than 0.5 and false discovery rate (FDR) less than 0.1 were considered differentially expressed [18]. The top differentially expressed genes from each dataset were visualized using pheatmap (version 1.0.12) with hierarchical clustering and Z-score normalization. Integrated analysis on ComBat-corrected data used limma (version 3.58.1) to identify high-confidence differentially expressed genes meeting stringent thresholds. Volcano plots were generated using EnhancedVolcano (version 1.20.0).

### 2.4. Meta-Analysis Integration

MetaVolcano (version 1.16.0) combined statistical evidence from three independent analyses through permutation testing (n_permutations = 10,000). Genes achieving significance in at least two of three datasets were considered high-confidence candidates. Correlation analysis used Pearson coefficients with hierarchical clustering to identify co-expression modules. Venn diagram analysis [19] was performed to identify dataset-specific and shared differentially expressed genes.

### 2.5. Machine Learning-Based Feature Selection

LASSO logistic regression [20] was performed using the glmnet package (version 4.1-8) with an L1 penalty (α = 1). The regularization parameter λ was selected by 10-fold cross-validation using cv.glmnet, and the value minimizing cross-validated deviance (λ_min_) was applied. Genes with non-zero coefficients were retained as candidate features. Random forest analysis was conducted using the randomForest package (version 4.7-1.1) [21] as an independent feature-ranking approach, with default parameters including 500 trees (ntree = 500) and internally optimized mtry values. Feature importance was quantified using the mean decrease in Gini index. LASSO-selected genes were further ranked by random forest importance, and genes with the highest combined scores were selected for signature development. The combined score was defined as the product of the absolute LASSO coefficient and the random forest importance (mean decrease in Gini). Model performance was evaluated using ROC analysis with predefined train/test splits and repeated stratified cross-validation. To minimize overfitting and information leakage, feature selection and model coefficients were fixed before validation, and only predefined gene signatures were evaluated during resampling.

### 2.6. Signature Score Development and Validation

A logistic regression model was constructed using the glm() function with family = binomial(link = “logit”). Maximum likelihood estimation optimized regression coefficients to maximize discriminative ability between responders and non-responders. The multi-gene signature score was calculated as the weighted sum of normalized expression values using regression coefficients (β_i_) derived from the logistic regression model:

Signature Score = Σ β_i_ × X_i_

where β_i_ represents the regression coefficient for gene i, and X_i_ denotes its normalized expression level. Model performance was evaluated through receiver operating characteristic (ROC) curve analysis using pROC (version 1.18.5) [22], calculating area under the curve (AUC) values for the signature score and individual genes. Bootstrap validation with 1000 iterations assessed model stability and generated 95% confidence intervals [23]. To further mitigate potential bias from a single data split, additional internal validation was conducted using repeated stratified cross-validation (5-fold cross-validation repeated 100 times). Importantly, feature selection and coefficient estimation were fixed prior to cross-validation, and only the predefined Signature Score was evaluated during resampling, thereby minimizing the risk of information leakage. Differences in Signature Score distributions between responders and non-responders were assessed using the Wilcoxon rank-sum test.

### 2.7. Pathway Enrichment Analysis

KEGG pathway enrichment analysis was performed using clusterProfiler (version 4.10.0) [24]. The enrichKEGG() function identified significantly enriched pathways (adjusted *p*-value less than 0.05) among the signature genes.

### 2.8. Statistical Analysis

All analyses were performed in R (version 4.5.0) (https://www.R-project.org/). Continuous variables were compared using Wilcoxon rank-sum test. Multiple testing correction applied the Benjamini–Hochberg method [25] to control FDR. All tests were two-sided with *p*-value less than 0.05 considered statistically significant. Data visualization used ggplot2 (version 3.5.0) [26].

## 3. Results

### 3.1. Data Integration Reveals Consistent Gene Expression Patterns Across Cohorts

**Figure 1:**
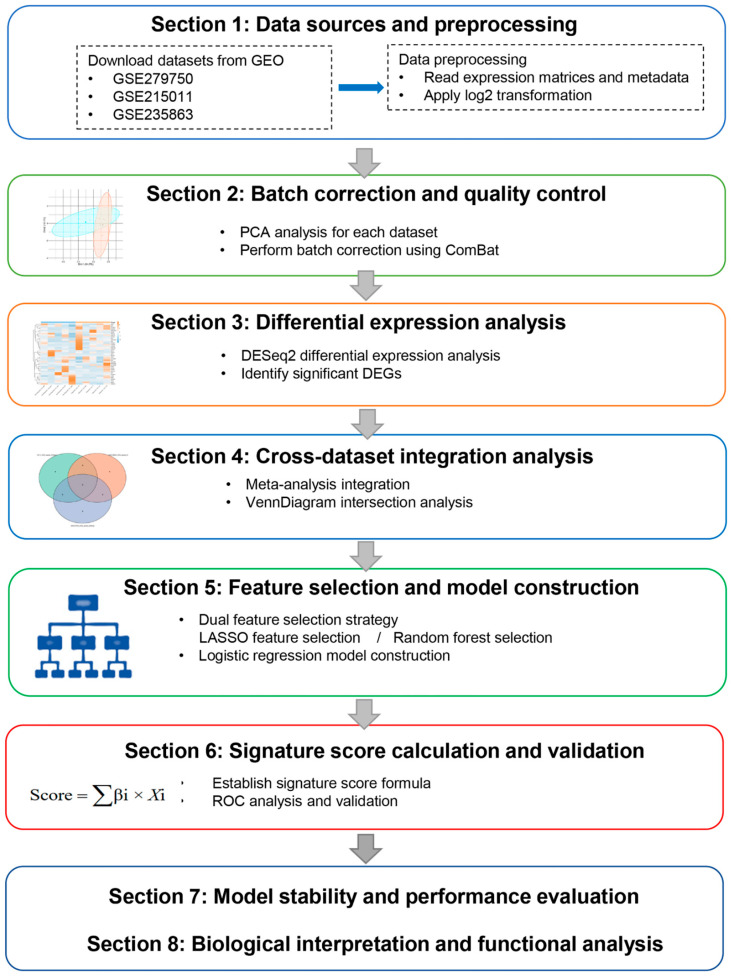
Integrated workflow for biomarker discovery and validation in immunotherapy response prediction. This study integrated three GEO datasets (GSE279750, GSE215011, GSE235863) to identify predictive biomarkers for immunotherapy response. The workflow comprised: (1) data acquisition and quality control; (2) batch effect correction and normalization; (3) differential expression analysis; (4) meta-analysis integration; (5) feature selection using LASSO and Random Forest; (6) signature score development and ROC validation; (7) model stability assessment via cross-validation, bootstrap, and external validation; (8) KEGG pathway enrichment analysis.

**Figure 2:**
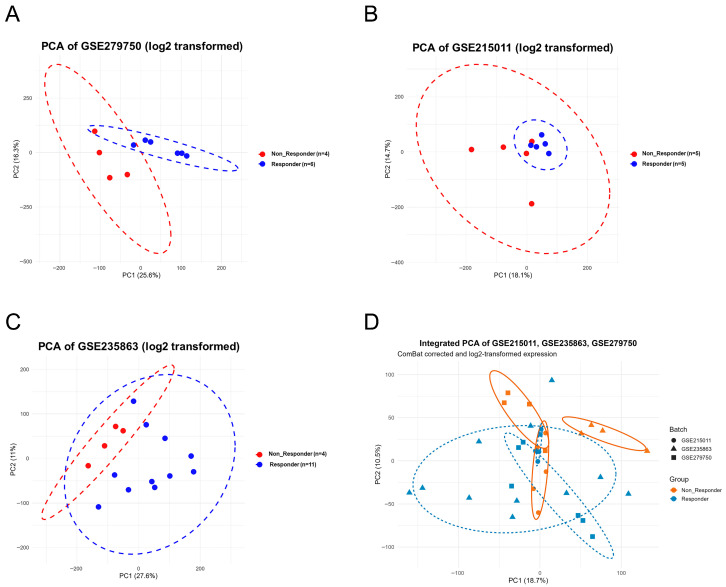
Principal component analysis of three datasets with batch effect correction. (**A**–**C**) PCA plots of log2-transformed expression data showing distribution of Responder (blue) and Non-Responder (red) samples across GSE279750 (*n* = 10), GSE215011 (*n* = 10), and GSE235863 (*n* = 15). Dashed ellipses represent 68% confidence intervals. (**D**) Integrated PCA of all 35 samples after ComBat batch correction, with different shapes indicating dataset origin (circle/triangle/square). Batch effects were effectively removed while maintaining clear separation between response groups (PC1: 18.7%, PC2: 10.5%).

To evaluate gene expression patterns and identify predictive biomarkers associated with immunotherapy response, we integrated gene expression data from three independent GEO cohorts comprising 35 patients with HCC treated with immune checkpoint inhibitors (GSE279750, *n* = 10; GSE215011, *n* = 10; GSE235863, *n* = 15). Across the integrated cohort, 22 patients were classified as responders and 13 as non-responders based on the original clinical annotations. These cohorts encompassed distinct but clinically relevant immunotherapy settings, including anti–PD-L1–based and anti–PD-1–based regimens, administered as monotherapy or in combination with lenvatinib, with tumor samples collected at different clinical time points. The integration workflow encompassed data acquisition, quality control, batch effect correction, differential expression analysis, meta-analysis integration, machine learning-based feature selection, signature score development, and biological pathway enrichment analysis (Figure 1). Principal component analysis (PCA) was performed on log2-transformed expression data to assess sample distribution and batch effects. Before correction, samples from the three datasets exhibited distinct clustering patterns, indicating significant batch effects (Figure 2A–C). PCA plots showed clear separation between Responder (blue) and Non-Responder (red) samples within each dataset. After applying ComBat batch effect correction, the integrated PCA plot demonstrated effective removal of technical variation while maintaining biological signal integrity (Figure 2D). The first two principal components explained 18.7% and 10.5% of total variance, respectively. Samples from different datasets (represented by circles, triangles, and squares) exhibited homogeneous distribution, presenting successful data integration. Importantly, batch correction preserved the biological separation between response groups, verifying successful multi-cohort harmonization while retaining signals essential for robust biomarker identification.

### 3.2. Differential Expression Analysis Identifies Response-Related Genes with Consistent Expression Patterns

**Figure 3:**
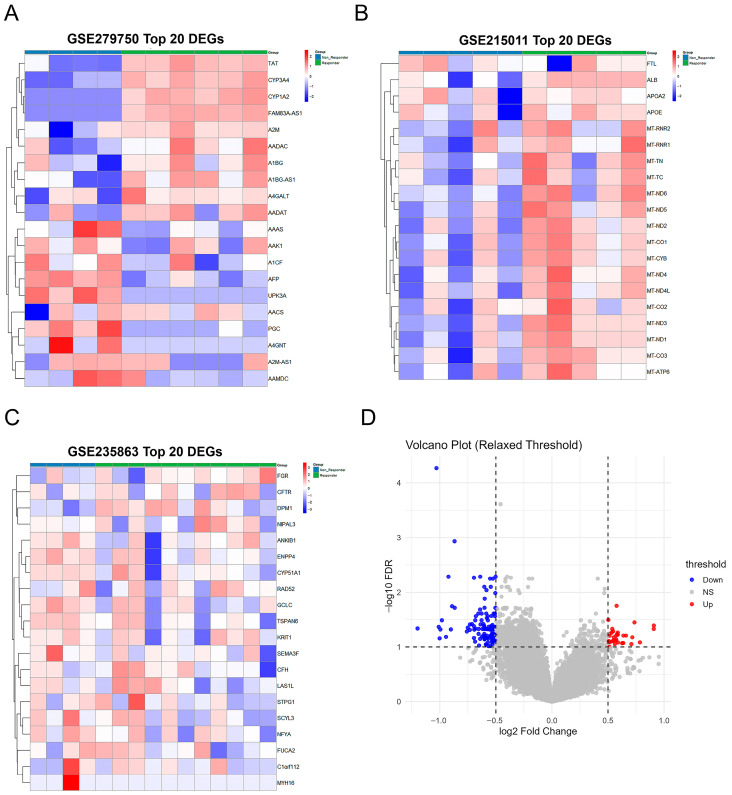
Differential gene expression analysis reveals distinct molecular signatures between treatment responders and non-responders. Comprehensive visualization of differentially expressed genes (DEGs) across three independent datasets and integrated analysis. (**A**) GSE279750 (*n* = 10) heatmap displays the top 20 DEGs with clear separation between Responder (green annotation) and Non-Responder (blue annotation) groups. Green and purple colors represent relative gene expression levels (Z-score normalized), with hierarchical clustering successfully distinguishing samples by treatment response status. (**B**) GSE215011 (*n* = 10) validates consistent differential expression patterns using its top 20 DEGs, where red indicates upregulation and blue indicates downregulation in responders versus non-responders. (**C**) GSE235863 (*n* = 15), the largest individual cohort, further substantiates reproducibility with its top 20 DEGs displayed in brown and teal colors. (**D**) Volcano plot of integrated analysis from ComBat-corrected data (*n* = 35 total) identifies 146 high-confidence DEGs (33 upregulated in red, 113 downregulated in blue) meeting stringent criteria (|log_2_FC| > 0.5, FDR < 0.1, indicated by dashed lines). Gray dots represent non-significant genes (NS).

We performed differential gene expression analysis independently on each dataset using DESeq2, identifying the top 20 differentially expressed genes (DEGs) in each cohort (Figure 3A–C). Heatmap visualizations revealed clear and consistent expression patterns between Responder and Non-Responder groups across all three datasets. In GSE279750, hierarchical clustering successfully separated samples by response status (Figure 3A); GSE215011 demonstrated similarly robust differential expression patterns (Figure 3B); while the largest cohort, GSE235863 (*n* = 15), further validated the consistency of these expression profiles (Figure 3C). Concordant results across three independent datasets strengthen DEG credibility, demonstrating biologically meaningful and reproducible expression patterns. Integrated analysis on ComBat-corrected data using limma identified 146 high-confidence DEGs (33 upregulated and 113 downregulated) meeting stringent thresholds (|log_2_FC| > 0.5, FDR < 0.1), as shown in the volcano plot (Figure 3D). These DEGs with consistent cross-cohort patterns provide a robust foundation for subsequent meta-analysis and machine learning-based biomarker identification.

### 3.3. Meta-Analysis Reveals Consistent High-Confidence Core Genes Across Datasets

**Figure 4:**
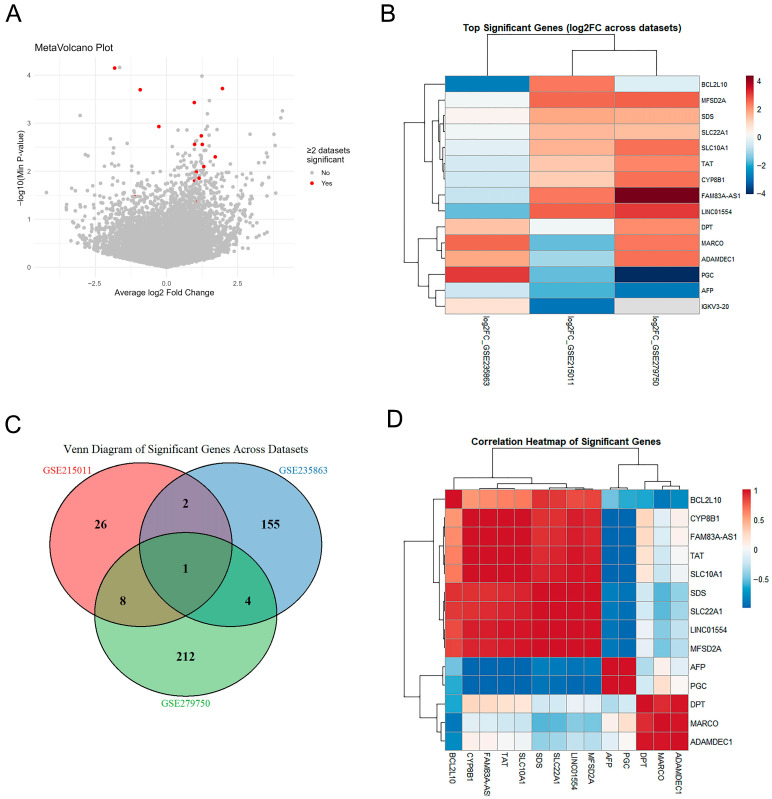
Meta-analysis identifies robust cross-dataset differentially expressed genes and their correlation patterns. Comprehensive meta-analysis integrating differential gene expression results from three independent datasets using MetaVolcano approach. (**A**) MetaVolcano plot displays genes based on average log_2_ fold change (x-axis) and cross-dataset significance (y-axis). Red dots (*n* = 15) indicate genes achieving statistical significance in at least two of three datasets, while gray dots represent non-significant genes. (**B**) Heatmap visualization of these 15 significant genes showing log_2_FC values across GSE215011, GSE235863, and GSE279750. Red colors indicate upregulation and blue colors indicate downregulation, demonstrating concordant expression patterns across all cohorts. (**C**) Venn diagram illustrating the overlap of significant genes among datasets. GSE279750 identified the largest number of unique DEGs (*n* = 225), while one core gene (LINC01554) was consistently significant across all three datasets. (**D**) Correlation heatmap of the 15 significant genes reveals co-expression patterns, with red indicating positive correlations and blue indicating negative correlations. Hierarchical clustering identifies distinct gene modules with coordinated expression changes, suggesting shared regulatory mechanisms underlying treatment response.

To identify genes with robust cross-dataset reproducibility, we employed MetaVolcano meta-analysis integrating statistical evidence from three independent cohorts. The MetaVolcano plot (Figure 4A) displays average log_2_ fold-change versus cross-dataset significance, identifying 15 genes achieving statistical significance in at least two of three datasets (red dots). Cross-dataset log_2_FC heatmaps (Figure 4B) of these 15 significant genes validated directional consistency across GSE215011, GSE235863, and GSE279750, with most genes maintaining uniform expression patterns. Venn diagram analysis (Figure 4C) revealed dataset-specific contributions across the three cohorts. GSE279750 identified the largest total number of DEGs (*n* = 225), with 212 unique DEGs specific to this dataset. GSE235863 identified 162 DEGs, and GSE215011 identified 37 DEGs. Notably, only one core gene (LINC01554) achieved statistical significance across all three datasets, highlighting the importance of meta-analysis for identifying reproducible markers. The correlation heatmap (Figure 4D) revealed co-expression patterns among the 15 significant genes, with hierarchical clustering identifying distinct gene modules suggesting coordinated regulatory mechanisms. Collectively, these results establish a robust set of cross-validated candidate genes, providing a reliable foundation for subsequent machine learning-based feature selection and predictive model development.

### 3.4. Machine Learning-Based Feature Selection Identifies Five Core Predictive Genes

**Figure 5:**
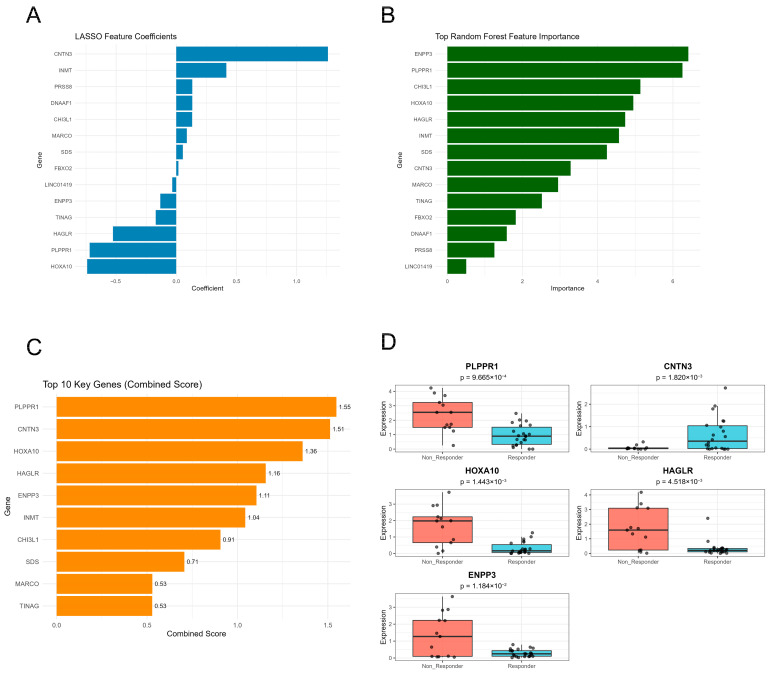
Machine learning-based feature selection identifies five core predictive genes for immunotherapy response. We employed two complementary machine learning approaches to identify the most informative features for predicting immunotherapy response. (**A**) LASSO (Least Absolute Shrinkage and Selection Operator) regression with L1 penalty performs automatic feature selection by shrinking coefficients of less important variables toward zero. The coefficient plot displays the magnitude and direction of coefficients for 14 candidate genes after cross-validation optimization. Blue bars represent positive coefficients, indicating genes positively associated with immunotherapy response, while negative coefficients (such as PLPPR1, HAGLR, HOXA10) are associated with Non-Responders. (**B**) Random forest analysis provides an independent assessment of feature importance through ensemble learning. Green bars indicate the mean decrease in Gini index for each gene across the forest, with higher values reflecting greater importance in classification. ENPP3, PLPPR1, and CHI3L1 exhibited the highest importance scores. (**C**) Combined score ranking integrates insights from both algorithms by multiplying the absolute LASSO coefficients by random forest importance. The top five genes (PLPPR1: 1.55, CNTN3: 1.51, HOXA10: 1.36, HAGLR: 1.16, ENPP3: 1.11) were selected for further validation. (**D**) Box plots demonstrate significant expression differences between Responders (blue) and Non-Responders (red) for all five genes (*p* < 0.05).

Importantly, genes identified by cross-dataset meta-analysis were used as candidates for feature selection; however, final model features were selected based on predictive contribution in multivariate machine learning models rather than overlap frequency alone. To identify the most informative features for predicting immunotherapy response, we employed two complementary machine learning approaches on the integrated expression matrix. LASSO regression with L1 penalty performs automatic feature selection by shrinking coefficients of less important variables toward zero (Figure 5A). Following cross-validation optimization, the coefficient plot displays the magnitude and direction of coefficients for 14 candidate genes selected based on predictive performance. Blue bars represent positive coefficients, indicating genes positively associated with immunotherapy response, while negative coefficients (PLPPR1, HAGLR, HOXA10) are associated with non-responders. Random forest analysis provides an independent assessment of feature importance through ensemble learning (Figure 5B). Green bars show the mean Gini decrease for each gene, with higher values indicating greater importance in classification decisions. ENPP3, PLPPR1, and CHI3L1 exhibited the highest random forest importance scores. To integrate insights from both algorithms, we calculated combined scores by multiplying the absolute LASSO coefficients by random forest importance (Figure 5C). Specifically, the combined score for each gene was defined as: Combined score = |β_LASSO_| × RF importance (mean decrease in Gini). Combined score ranking identified the top five genes: PLPPR1 (1.55), CNTN3 (1.51), HOXA10 (1.36), HAGLR (1.16), and ENPP3 (1.11), which were selected for further validation. Box plot analysis demonstrated significant expression differences between responders (blue) and non-responders (red) for all five genes (*p* < 0.05) (Figure 5D). To evaluate the predictive performance, we constructed a logistic regression model based on the five-gene signature and first assessed its discriminative ability using a predefined training and testing split. ROC analysis demonstrated strong discriminative performance in the initial training/testing evaluation (Figure S1A). Given the limited sample size, we further evaluated the stability of this result using repeated stratified cross-validation with a fixed Signature Score. Across resampling iterations, the model showed highly consistent discriminative performance, yielding consistently high AUC values (Figure S1B,C). Collectively, these results establish a robust five-gene predictive signature derived from dual-algorithm feature selection, supported by consistent performance across independent testing and repeated resampling, underscoring the internal robustness of the integrated machine learning framework for predicting immunotherapy response within the current cohort.

### 3.5. The Five-Gene Signature Score Demonstrates Consistent Predictive Performance with Internal Stability

Based on the machine learning–based feature selection results, the top five genes (PLPPR1, CNTN3, HOXA10, HAGLR, and ENPP3) were incorporated into a logistic regression model. Using the integrated transcriptomic dataset, regression coefficients were optimized through maximum likelihood estimation to model discrimination between responders and non-responders. The resulting five-gene signature score was defined as follows:

Signature Score = (−194.836 × PLPPR1) + (147.927 × CNTN3) − (326.820 × HOXA10) − (2.582 × HAGLR) − (41.937 × ENPP3)

**Figure 6:**
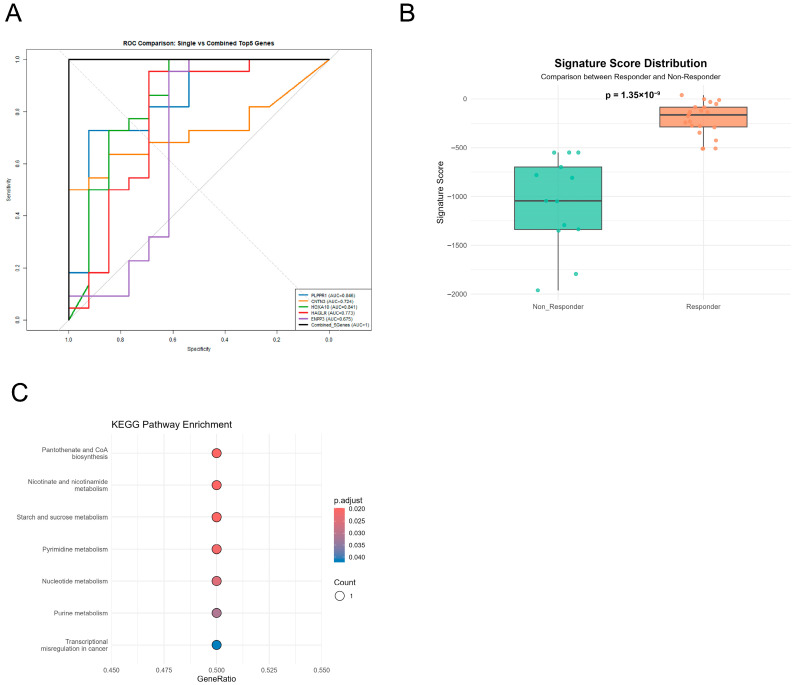
Development, validation, and biological interpretation of the five-gene signature prediction model. (**A**) ROC comparison of single vs. combined models. ROC analysis demonstrated that the combined five-gene model (AUC = 1.0) outperformed individual genes (PLPPR1: 0.846; CNTN3: 0.724; HOXA10: 0.841; HAGLR: 0.773; ENPP3: 0.675). (**B**) Signature Score distribution shows complete separation between Responder and Non-Responder groups (*p* = 1.35 × 10^−9^, Wilcoxon test), with Responders exhibiting significantly higher scores than Non-Responders. (**C**) KEGG pathway enrichment analysis shows significant enrichment in metabolism-related pathways (pantothenate and CoA biosynthesis, nicotinate and nicotinamide metabolism, nucleotide metabolism) and transcriptional misregulation in cancer (*p* < 0.05), suggesting these core genes may influence immunotherapy response through metabolic reprogramming.

In this model, the positive coefficient of CNTN3 indicates a positive association between its expression and immunotherapy response, whereas HOXA10 exhibited the largest negative coefficient, identifying it as a major contributor to non-response. PLPPR1 and ENPP3 also showed negative associations with response, while HAGLR contributed a smaller effect. The absolute coefficient magnitudes reflect the relative contributions of individual genes to the composite score. Receiver operating characteristic (ROC) analysis showed that the five-gene signature score achieved complete separation between responders and non-responders in the integrated cohort, yielding an observed AUC of 1.0 (Figure 6A), exceeding individual gene performance (AUC range: 0.675–0.841). Consistent with this, box plot visualization demonstrated distinct signature score distributions between groups without overlap (*p* = 1.35 × 10^−9^, Wilcoxon rank-sum test; Figure 6B). To assess internal stability, bootstrap validation with 1000 resampling iterations was performed (Figure S1D). The bootstrap AUC distribution showed minimal variation, with a mean AUC of 1.0 and a 95% confidence interval of [1.0, 1.0], indicating stable performance within the current dataset. Given the limited sample size, these results should be interpreted as evidence of internal consistency rather than definitive generalizability.

### 3.6. Pathway Enrichment Analysis Reveals Metabolic Reprogramming as a Key Mechanism

To elucidate biological mechanisms underlying the five-gene signature’s predictive power, we performed KEGG pathway enrichment analysis using clusterProfiler (Figure 6C). Overrepresentation analysis identified seven significantly enriched pathways (corrected *p* < 0.05, Benjamini–Hochberg correction). Core genes were significantly enriched in metabolism-related pathways, including pantothenate and coenzyme A biosynthesis, nicotinic acid and nicotinamide metabolism, starch and sucrose metabolism, pyrimidine metabolism, nucleotide metabolism, and purine metabolism. Additionally, enrichment in transcriptional dysregulation in cancer suggests involvement in oncogenic networks. These findings reveal that metabolic reprogramming represents a key determinant of immunotherapy response, with the five core genes coordinately regulating cellular energy metabolism, nucleotide biosynthesis, and redox homeostasis, thereby influencing tumor microenvironment and immune cell function.

## 4. Discussion

Our study developed and internally evaluated a five-gene signature (PLPPR1, CNTN3, HOXA10, HAGLR, and ENPP3) for predicting immunotherapy response through integrative analysis of three independent GEO transcriptomic datasets. Within the combined cohort, the signature showed high discriminative ability and internally stable performance across resampling-based validation analyses. However, given the limited sample size and retrospective design, these findings should be interpreted with caution. Further validation in larger, independent, and prospectively collected clinical cohorts is required to establish robustness, generalizability, and clinical applicability.

The identification of metabolic reprogramming as a key mechanism underlying immunotherapy response aligns with emerging evidence highlighting the critical role of metabolism in immune function and tumor microenvironment modulation [27,28]. Our pathway enrichment analysis revealed significant involvement of nucleotide metabolism, coenzyme biosynthesis, and energy metabolism pathways, consistent with findings that metabolic alterations profoundly impact immune cell infiltration and activation [29,30]. To further explore gene-level contributions within the signature, our integrated LASSO-random forest approach achieved near-perfect classification through robust cross-validation while minimizing overfitting and maximizing biological interpretability. Within this framework, HOXA10 emerged as the strongest negative predictor of immunotherapy response (coefficient: −326.820), consistent with its role as a master transcriptional regulator implicated in immune evasion and treatment resistance [31,32]. High HOXA10 levels may promote immunosuppressive microenvironments by regulating immune cell differentiation [33,34]. CNTN3, with positive coefficient (+147.927), encodes a cell adhesion molecule that may facilitate beneficial immune cell infiltration or enhance immune recognition mechanisms [35]. PLPPR1 encodes a membrane-associated phospholipid phosphatase–related protein involved in lipid signaling and membrane dynamics, processes increasingly recognized as critical for immune receptor signaling, immune cell activation, and metabolic fitness within the tumor microenvironment [36,37]. HAGLR (also known as HOXD-AS1), a long non-coding RNA, has been implicated in transcriptional regulation linked to tumor progression, epithelial–mesenchymal transition, and immune suppression, suggesting a role in shaping immunosuppressive tumor states that may limit immunotherapy efficacy [38,39]. ENPP3 has recently been identified as an extracellular cGAMP hydrolase that acts as an innate immune checkpoint by attenuating cGAMP–STING signaling [40]. Genetic or functional disruption of ENPP3 enhances antitumor immunity in a STING-dependent manner, implicating ENPP3 in immune regulation within the tumor microenvironment [40]. Collectively, these genes converge on metabolic regulation, transcriptional control, and immune modulation, key biological processes underlying immunotherapy responsiveness.

Moreover, our meta-analysis integrating three independent cohorts demonstrated biomarker reproducibility across distinct populations. Notably, only LINC01554 achieved statistical significance across all three datasets, underscoring the critical importance of meta-analytical approaches for the discovery of robust biomarkers. Previous studies have identified LINC01554 as a liver-enriched tumor suppressor lncRNA that regulates glucose metabolism by promoting PKM2 degradation and inhibiting the Akt/mTOR pathway, thereby suppressing HCC progression [41]. Its downregulation correlates with larger tumor size, advanced TNM stage, and poorer prognosis in HCC [41]. Moreover, recent integrative models have consistently recognized LINC01554 as a protective, metabolism-related lncRNA linked to favorable immunotherapy response [42]. These findings support LINC01554 as a metabolic–immune regulatory biomarker with translational potential in HCC. Pathway analysis revealed metabolic enrichment alongside regulatory insights from HOXA10 and CNTN3, highlighting the integrated contribution of metabolic and transcriptional regulation to immunotherapy outcomes in HCC and suggesting therapeutic targets for combination strategies where metabolic modulators could enhance immunotherapy efficacy.

### Study Limitations and Future Directions

Several limitations should be acknowledged. First, the integrated cohort size was relatively small (*n* = 35), which may increase the risk of optimistic performance estimates despite internal validation. Although repeated stratified cross-validation with a fixed Signature Score was applied to mitigate overfitting and information leakage, the high AUC values observed should be interpreted as evidence of internal stability rather than generalizability. Therefore, external validation in larger, independent, and prospectively collected cohorts is required to confirm clinical utility. In addition, while bulk transcriptomic analysis enabled identification of a metabolically relevant signature, future single-cell or spatial transcriptomic studies may provide deeper mechanistic insights and enhance translational relevance for precision immunotherapy.

## 5. Conclusions

In conclusion, this study identified and internally evaluated a five-gene metabolic signature (PLPPR1, CNTN3, HOXA10, HAGLR, and ENPP3) associated with immunotherapy response in hepatocellular carcinoma through integrative analysis of multiple transcriptomic datasets. By combining meta-analysis with complementary machine learning approaches, we demonstrated internally consistent discrimination between responders and non-responders and provided biologically interpretable insights into metabolic and immune-related mechanisms. While these findings offer preliminary evidence supporting the potential utility of this signature for response stratification, external validation in larger, independent, and prospectively collected cohorts will be essential before clinical translation. Our study highlights the value of integrative multi-cohort and systems-level approaches for biomarker discovery in immuno-oncology.

## Data Availability

The data presented in this study are openly available in GEO at https://www.ncbi.nlm.nih.gov/geo/, accessed on 1 August 2025, reference number [GSE215011, GSE235863, and GSE279750].

## Supplementary Materials

The following supporting information can be downloaded at: https://www.mdpi.com/article/10.3390/diagnostics16010085/s1, Figure S1: Robust internal validation of the five gene Signature Score for predicting immunotherapy response.
