# Supplementary material for: Biomarker-Based Precision Prediction of Immunotherapy Response in Hepatocellular Carcinoma
Source: Diagnostics (Basel). 2025 Dec 26;16(1):85. doi: 10.3390/diagnostics16010085 (PMC12786039; doi:10.3390/diagnostics16010085)
Supplement: Supplementary file 1 [file diagnostics-16-00085-s001.zip › diagnostics-3986787-supplementary.pdf]

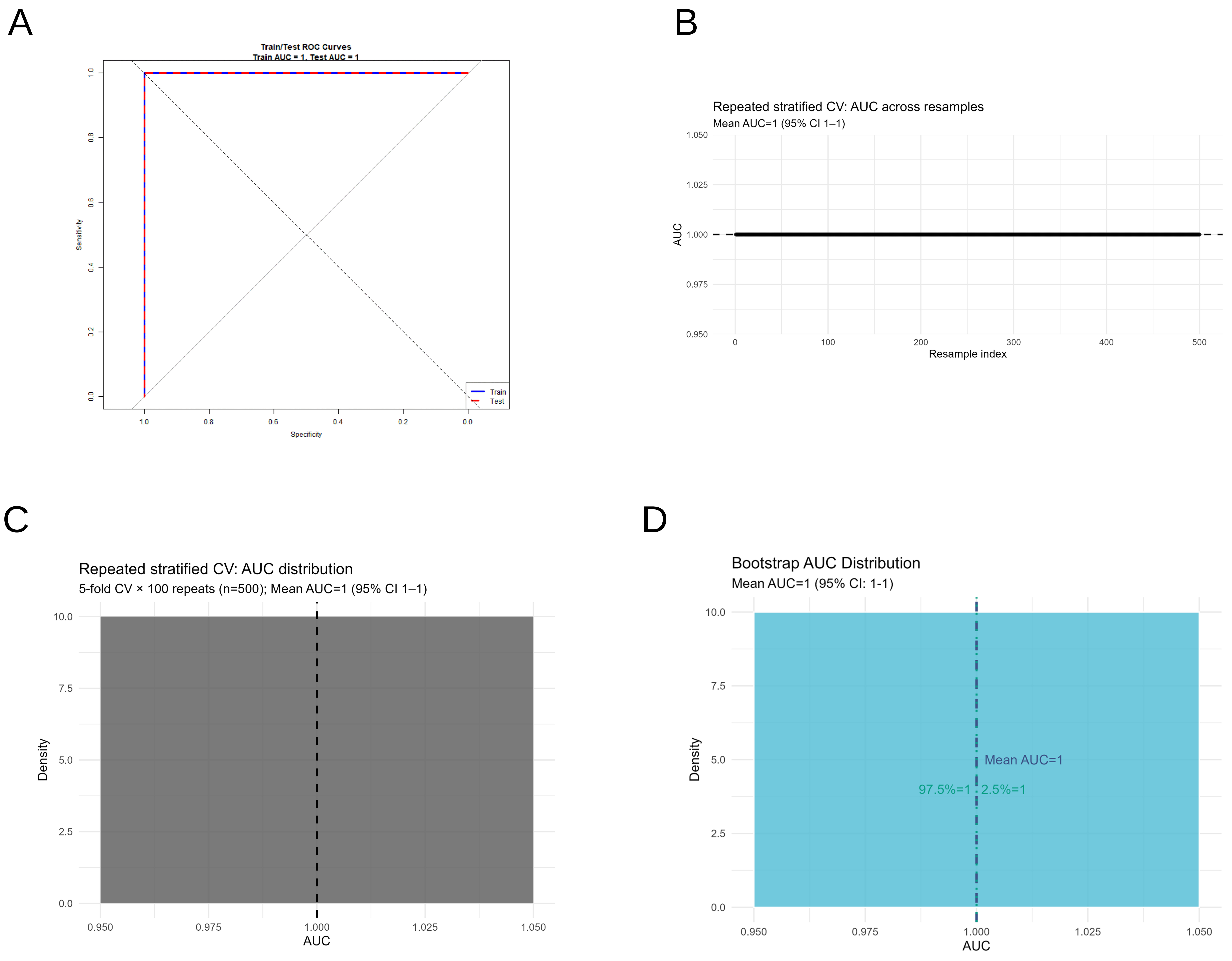

Figure S1. Robust internal validation of the five-gene Signature Score for predicting immunotherapy response. (A) Receiver operating characteristic (ROC) curves showing perfect classification performance (AUC = 1.0) for both training (blue) and testing (red) sets using the predefined Signature Score.(B) Stability of Signature Score performance evaluated by repeated stratified cross-validation, where the area under the ROC curve (AUC) is shown across all resampled folds (5-fold cross-validation repeated 100 times; n = 500 resamples). (C) Distribution of AUC values obtained from repeated stratified cross-validation, demonstrating a mean AUC of 1.0 with a 95% confidence interval of 1.0–1.0, indicating consistent out-of-fold classification performance across resamples. (D) Bootstrap stability validation (1000 resampling iterations) reveals highly stable model performance (Mean AUC = 1.0, 95% CI: 1.0-1.0), with both 97.5% and 2.5% percentiles at 1.0, demonstrating excellent reproducibility.
